# “Precision” Medicine in Membranous Nephropathy: Serology-Guided Therapy

**DOI:** 10.1016/j.ekir.2023.01.024

**Published:** 2023-01-28

**Authors:** Richard J. Glassock, Fernando C. Fervenza

**Affiliations:** 1 Department of Medicine, Geffen School of Medicine at UCLA, Los Angeles, California, USA; 2 Department of Medicine, Division of Nephrology and Hypertension, Mayo Clinic, Rochester, Minnesota, USA

See Clinical Research on Page 432

The epoch-defining discovery of the role of autoantibodies to M-type phospholipase A2 receptor (anti-PLA2R) in most cases of apparently primary membranous nephropathy (MN) by Beck *et al.*^1^ in 2009 triggered a revolution in diagnosis and management of this condition. The prior “one size fits all” approach to MN was no longer tenable. Soon after, a variety of studies strongly suggested that measurement and quantification of anti-PLA2R antibodies could have an important impact on the diagnosis and therapy of MN^2^^,^^3^ and approved assays quickly appeared on the scene, namely, a quantitative enzyme-linked immunoadsorbent assay (ELISA) and a semiquantitative indirect immunofluorescent assay (IFA).^4^ In 2017, a proposal was first formally articulated, which incorporated the ELISA measurement of anti-PLA2R antibodies into an algorithm of “precision” therapy for apparently primary MN.^5^ In 2021, the Kidney Disease: Improving Global Outcomes Clinical Practice Guidelines for management of MN fully embraced the importance of serologic investigation of MN (using anti-PLA2R by ELISA and IFA) for both diagnosis and therapy of MN.^6^ Concomitantly, the spectrum of antigen-antibody systems in apparently primary MN were expanded well beyond the seminal anti-PLA2R paradigm.^7^ However, actual clinical evaluation of the benefits of serology-guided treatment regimens lagged behind the proposals and practice points embedded in algorithms and guidelines.

This gap has now been filled, at least in part, by a single center, prospective, single arm cohort study from the Radboud University Medical Center in Nijmegen, Netherlands, a world–renowned unit with a long history of impactful clinical research in MN.^8^ Vink and colleagues describe the results of a serology-guided protocol for therapy of anti-PLA2R antibody positive MN (*n* = 65) compared to a historical control group treated in a serologically agnostic fashion using daily cyclophosphamide and steroids (*n* = 40). The study was initiated in 2013 and completed in 2019, before the publication of the Kidney Disease: Improving Global Outcomes-Glomerulonephritis Clinical Practice Guidelines in 2021.^6^ It is very important to understand and appreciate the specific details of the protocol because they lead to issues in its interpretation and clinical application, in our opinion.

All of the patients studied had anti-PLA2R mediated MN (biopsy proven in 92%) with nephrotic syndrome and an estimated glomerular filtration rate of <60 ml/min per 1.73 m^2^ was present in 65% of the subjects enrolled in the serology-guided protocol (average 46 ml/min per 1.73 m^2^). The MN was incident (newly diagnosed) in 77% and prevalent (relapsing or treatment resistant) in 23% of the subjects examined. The anti-PLA2R antibody was measured exclusively by IFA, not ELISA, and was considered either positive or negative, in a nonquantitative manner, with indeterminate samples considered as positive. The enrolled subjects all were deemed to be at high or very high risk of progression on the basis of well-established clinical and laboratory criteria, used by the center for decades. Kidney biopsy information was not used to define the risk of progression, and rebiopsy was not incorporated into the protocol. The 23% prevalent patients had received a variety of prior immunosuppressant therapies and the 67% incident patients were naïve to immunosuppressant therapy. The serology-guided therapy subjects had a short interval between diagnosis and therapy (about 5 months), whereas the historical control cohort has a slightly longer interval of about 8 months. The historical control group included 27% anti-PLA2R negative subjects, whereas 100% of the serology-guided therapy cohort were anti-PLA2R positive (by IFA). Otherwise, the serology-guided and historical control groups were reasonably comparable.

The serology-guided treatment protocol is shown in Figure 1. Oral cyclophosphamide (CYC; 1.5 mg/kg per day) plus i.v. pulse-methyl prednisolone (IVMP) pulses followed by low-dose oral steroids were used exclusively in both the groups. No patient in either group received rituximab or a calcineurin inhibitor during the protocol-driven phase of the study. Prophylaxis for pneumocystis was apparently not employed routinely. If the anti-PLA2R antibody converted to negative (by IFA) by 8 weeks (an immunologic remission), the CYC was stopped, steroids were rapidly tapered and the patient was observed for immunologic relapse and/or clinical remission (complete or partial). In the absence of an immunologic remission at 8 weeks, CYC and oral steroids were continued for another 8 weeks and IV pulse MP was re-administered. Again, if an immunologic remission was achieved, the CYC was stopped, and the steroids tapered. If no immunologic remission at 16 weeks was evident, the cycle of IV MP was again repeated, and CYC and steroid continued for another 8 weeks (total 24 weeks). If no immunologic remission was obtained after 24 weeks, the remaining subjects were treated with oral mycophenolate mofetil (2.0 g/d) and CYC was stopped permanently. Treatment regimens were repeated for full relapses. The historical control group received an initial IVMP and continuous oral CYC for 6 months with low-dose oral steroids.

**Figure 1:**
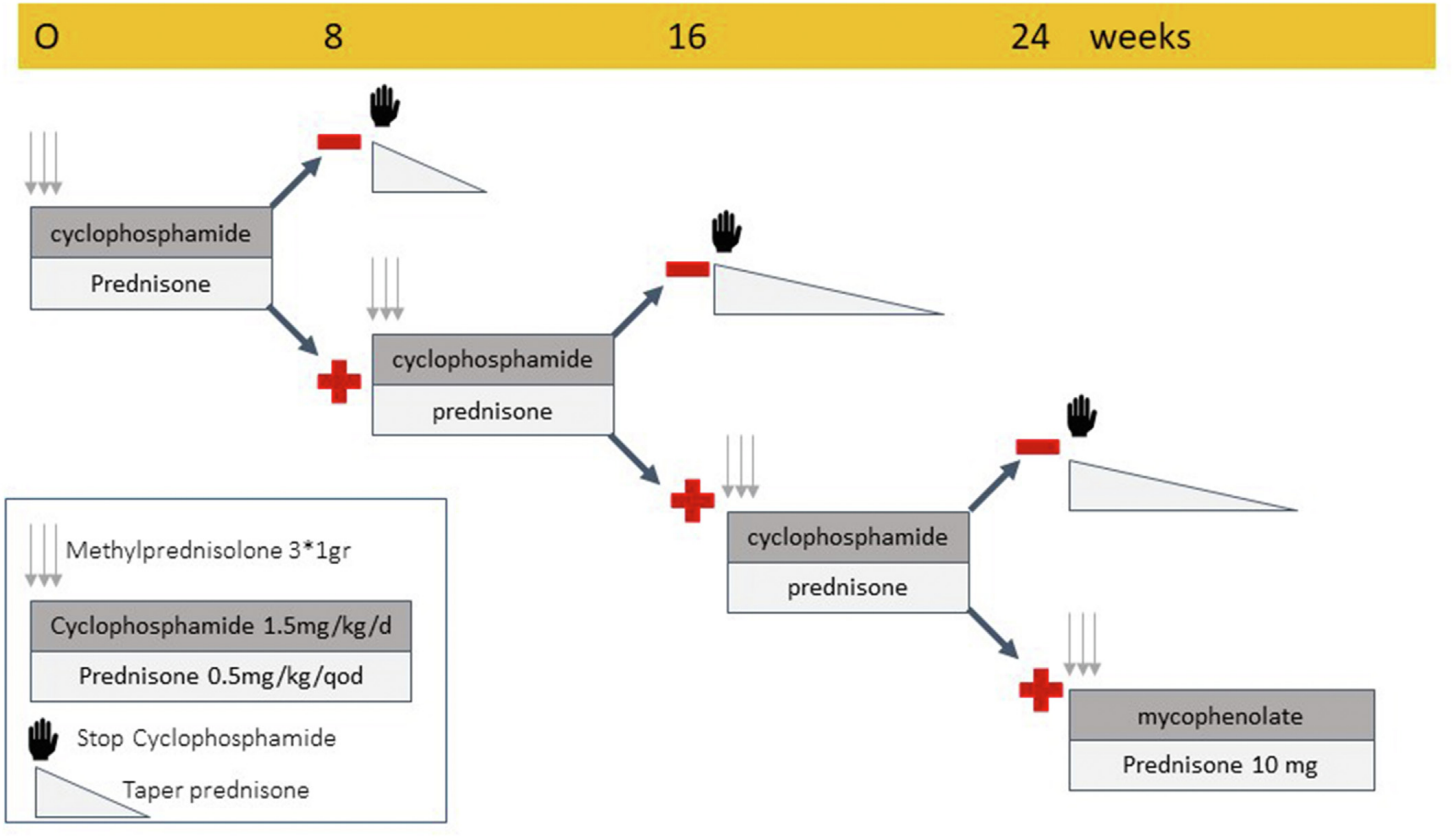
Serology-guided protocol for therapy of phospholipase A2 receptor antibody positive membranous nephropathy (from Vink C *et al.*^8^ KI Reports, 2022, this issue. Supplementary Figure 1)

The primary outcome parameter examined was the cumulative occurrence of complete and partial clinical remission at 24 months (96 weeks), which persisted for at least 6 months after the first course of serology-guided therapy. A variety of secondary end-points, including clinical and immunologic remissions and relapses occurring during the treatment protocol period, the total duration of immunosuppressive therapy, the cumulative total dosage of CYC, kidney function deterioration, and adverse events were also examined.

The most important finding was that 42 of the 65 (64%) patients enrolled in the serology-guided protocol developed a clinical remission (partial or complete; 27 at 8 weeks, 8 at 16 weeks, and 7 at 24 weeks). The cumulative remission rate at 24 months (including the period of mycophenolate mofetil administration) was 92%. Complete immunologic remission occurred in 71% by 8 weeks; in 86% at 16 weeks, in 88% at 24 weeks, and in 92% at 36 months. The clinical remission outcomes were quite similar to those experienced in the historical control cohort. However, the cumulative dosage of CYC was only 11.8 g in the serology-guided cohort compared to 18.9 g in the historical cohort. The relapse rates during treatment were also quite similar between the serology-guided cohort and the historical controls but both rates were higher than that observed in a cohort with more prolonged CYC therapy, suggesting that a higher relapse rate is a potential disadvantage of the less intensive CYC exposure. Surprisingly, adverse events were fairly common in the serology-guided cohort as follows: 40% infections, 12% leucopenia, and 17% hyperglycemia. This may have been due to the use of repeated IVMP treatments. No comparison of adverse events in the historical controls was made available.

Discordancies between immunologic remission and clinical remission were observed in 8 patients (12% of the entire cohort or 1 in every 8 patients); 4 with clinical remission despite positive IFA, 2 with persistent proteinuria without immunologic relapse, and 2 with relapse of proteinuria and negative IFA. The mechanisms responsible for these discordant results remains uncertain largely because of the lack of repeat biopsy (secondary changes from basement membrane and podocyte remodeling, focal sclerosis, or interstitial damage may result in indefinite persistence of low-level proteinuria in the presence of immunologic remission), and exclusive reliance on an IFA test for determining immunologic remission, which cannot rule out the possibility that these patients had become anti-PLA2R negative when tested on an ELISA assay. The conclusion that serology-guided therapy using an IFA testing protocol and oral CYC, IVMP, and low-dose steroids is effective in PLA2R associated primary MN and permits a lower cumulative exposure to CYC is fully warranted by these observations. In an 80 kg patient, an 8-week course of this therapy corresponds to a total CYC cumulative dose of 7.2 g, which is well below the Kidney Disease: Improving Global Outcomes recommendations to limit CYC dose to 25 g total.^6^ However, it should be noted that only 27 (42%) of the patients had long-lasting remission after only 8 weeks of therapy, with an additional 15 patients requiring 16 to 24 weeks of therapy.

This is an important contribution to the burgeoning science of “precision” medicine in the domain of PLA2R-associated primary MN. Nevertheless, it contains several weaknesses that may limit its widespread application. As the authors acknowledge, it is a single-center study that is not randomized or controlled. The historical controls used may not have been comparable to the serology-guided cohort in some crucial way, causing a bias. More importantly, the use of a semiquantitative IFA for determining immunologic activity instead of the more quantitative ELISA makes it impossible to judge whether a reduction in antibody levels, short of full disappearance of antibody, could be used to make treatment decisions (as suggested in the 2017 algorithm mentioned above).^5^ Of course, the protocol used provides information applicable only to a daily CYC regimen accompanied IVMP and low-dose oral steroids. Direct Comparisons of this regimen to rituximab-based therapy, which is far less toxic, or to combinations of rituximab and CYC, are needed to more fully understand the advantages and disadvantages of a serology-guided treatment paradigm for primary PLA2R associated MN. The Rituximab or Cyclosporine for Membranous Nephropathy trial^9^showed that rituximab (1 g, 2 doses, repeated at 6 months) without steroids resulted in 60% of the patients achieving a clinical remission at 24 months. However, this apparent suboptimal response can be explained by the trial protocol defining treatment failure as <25% reduction of proteinuria at 6 months. When rituximab was used without the constrains of a clinical trial, remission rates of 80% at 24 months was achieved.^S1^

Obviously, none of these data can be applied to the very heterogeneous population of patients with non-PLA2 associated apparently primary MN. The adverse event profile of the serology-guided regimen described here is somewhat disconcerting, despite the very welcome decrease in CYC cumulative dose exposure. The possibility that these adverse events were driven by the high dose steroid component (up to a total of 9 g of IVMP in some patients) of the regimen is worthy of consideration. The clinico-immunologic discordancies observed are interesting but presently lack a mechanistic explanation. Finally, it is worth emphasizing that this protocol of serology-guided CYC plus steroids was carried out only in high risk to very high risk PLA2R antibody associated MN, defined exclusively by clinical features, not including the quantitative level of circulating antibody, which is only discernable by use of ELISA testing. It is also surprising that no data regarding estimated glomerular filtration rate on follow-up are provided. Achievement of clinical remission should equate to long-term maintenance or improvement of kidney function.

Despite its limitations, the authors should be lauded for beginning to travel on the long road to “precision” medicine for the pattern-of-injury lesion of MN. Clearly, they show that the “one size fits all” treatment approach can and should be challenged. Hopefully, this novel study will spark greater interest in serology-driven regimens for MN already heralded by the seminal proposal made in 2017.

## Disclosure

RJG discloses that he is a compensated consultant to Alexion, Omeros, Travere, BioCryst, Equillium, Aurini, Ionis, RenaSight, Novartis, River3Renal, Otsuka, Arrowhead, Chinook, and Vera. He also receives an editorial stipend from Wolters-Kluwer as an Associate Editor for UpToDate. FCF discloses that he is a consultant to Genentech and an Associate Editor for UpToDate.
